# Shorter Anogenital Distance Predicts Poorer Semen Quality in Young Men in Rochester, New York

**DOI:** 10.1289/ehp.1103421

**Published:** 2011-03-04

**Authors:** Jaime Mendiola, Richard W. Stahlhut, Niels Jørgensen, Fan Liu, Shanna H. Swan

**Affiliations:** 1 Department of Obstetrics and Gynecology, School of Medicine and Dentistry, University of Rochester, Rochester, New York, USA; 2 Division of Preventive Medicine and Public Health, School of Medicine, University of Murcia, Murcia, Espinardo, Spain; 3 University Department of Growth and Reproduction, University of Copenhagen, Rigshospitalet, Copenhagen, Denmark; 4 Mount Sinai School of Medicine, New York, New York, USA

**Keywords:** anogenital distance, antiandrogens, endocrine disruption, semen quality, testicular dysgenesis

## Abstract

Background: In male rodents, anogenital distance (AGD) provides a sensitive and continuous correlate of androgen exposure in the intrauterine environment and predicts later reproductive success. Some endocrine-disrupting chemicals can alter male reproductive tract development, including shortening AGD, in both rodents and humans. Whether AGD is related to semen quality in human is unknown.

Objective: We examined associations between AGD and semen parameters in adult males.

Methods: We used multiple regression analyses to model the relationships between sperm parameters and two alternative measures of AGD [from the anus to the posterior base of the scrotum (AGD_AS_) and to the cephalad insertion of the penis (AGD_AP_)] in 126 volunteers in Rochester, New York.

Results: AGD_AS_, but not AGD_AP_, was associated with sperm concentration, motility, morphology, total sperm count, and total motile count (*p*-values, 0.002–0.048). Men with AGD_AS_ below (vs. above) the median were 7.3 times more likely (95% confidence interval, 2.5–21.6) to have a low sperm concentration (< 20 × 10^6^/mL). For a typical study participant, sperm concentrations were 34.7 × 10^6^/mL and 51.6 × 10^6^/mL at the 25th and 75th percentiles of (adjusted) AGD_AS_.

Conclusions: In our population, AGD_AS_ was a strong correlate of all semen parameters and a predictor of low sperm concentration. In animals, male AGD at birth reflects androgen levels during the masculinization programming window and predicts adult AGD and reproductive function. Our results suggest, therefore, that the androgenic environment during early fetal life exerts a fundamental influence on both AGD and adult sperm counts in humans, as demonstrated in rodents.

A wide range of environmental chemicals can interfere with androgen production and signaling and have been shown to alter the development of the male reproductive tract in experimental animals (Foster 2006; Gray et al. 2006). Establishing links between antiandrogenic exposure *in utero* and similar outcomes in humans is challenging, however, in part because the genital anomalies traditionally examined in humans (e.g., hypospadias) occur with such a low incidence that studying them requires very large populations. Thus, a more sensitive (and continuous) measure of the developmental androgenic milieu is desirable.

Anogenital distance (AGD; distance from anus to genitals) may serve as such a measure. AGD is routinely used in animal toxicology studies and is the developmental end point most sensitive to antiandrogenic exposure. In rodents and other mammals, AGD has been shown to reflect the amount of androgen to which a male fetus is exposed in early development; higher *in utero* androgen exposure results in longer and more masculine AGD. Recent interest in reproductive effects of antiandrogens has focused on the phthalates, particularly diethylhexyl phthalate (DEHP) and dibutyl phthalate (DBP), whose antiandrogenic effects have been directly demonstrated in rodent models (Gray et al. 2006; Scott et al. 2008). We previously reported strong inverse associations between prenatal phthalate exposure (particularly DEHP and DBP) and shorter male AGD in human infants (Swan 2008; Swan et al. 2005).

In many rodent studies, shortened AGD is seen in conjunction with frank defects such as hypospadias and cryptorchidism, and shorter AGD has been seen in conjunction with hypospadias in human males (Hsieh et al. 2008). Moreover, in male rodents, shortened (weight-adjusted) AGD persists into adulthood (Hotchkiss et al. 2004) and predicts compromised reproductive function in the mature male (Macleod et al. 2010; Scott et al. 2008). However, to our knowledge, no study has examined associations between AGD in adults and sperm number or quality.

Definitively demonstrating that AGD provides a link between prenatal antiandrogen exposure and adult reproductive function in humans would require the availability of biological samples reflecting prenatal exposure and subsequent follow-up across the many years between exposure and sexual maturation. However, associations between AGD and adult reproductive function in humans would provide indirect evidence.

In this study, we explored the hypothesis that AGD may be a predictor of semen quality in adult humans. If confirmed, this biomarker may provide information about the androgenic hormonal milieu during fetal development and may be useful in studies of reproductive development and function in adulthood.

## Materials and Methods

*Study population.* Subjects were participants in the Rochester Young Men’s Study (RYMS), a cross-sectional study of young men conducted in 2009–2010 at the University of Rochester (Rochester, NY). RYMS is part of an international study funded by the European Union Seventh Framework Program (Environment), “Developmental Effects of Environment on Reproductive Health” (DEER). Men were recruited into RYMS through flyers and newspapers at college and university campuses in the Rochester area. Subjects were eligible if they were born in the United States after 31 December 1987, able to read and speak English, and able to contact their mother and ask her to complete a questionnaire. In response to advertisements placed at local colleges, a total of 389 potential participants contacted our study coordinator between spring 2009 and spring 2010. Of these, 305 (78.4%) met all eligibility criteria, and 222 men participated in the study. AGD measurements were obtained only for men who enrolled September 2009 and later. One man with a history of testicular cancer was azospermic (sperm count of 0) and was not included in analysis. Motility data were excluded for one man whose time to semen analysis exceeded 30 min. The analysis reported here includes all 126 men with complete data on all study outcomes and covariates, including both measures of AGD, except sperm morphology data, which was available for only 124 men.

The study included a physical examination; blood, urine, and semen samples; and completion of a brief questionnaire. Subjects received $75 upon completion of all study components. Data from the mother’s questionnaire were not considered in this analysis. The University of Rochester Research Subjects Review Board approved the study, and written informed consent was obtained from all subjects before their participation.

*Semen collection and analysis.* Men collected semen samples by masturbation at the clinic and were asked to report the time of their previous ejaculation. Although they were asked to abstain from ejaculation for at least 48 hr before sample collection, they were not excluded if they had not. Abstinence times reported to be > 240 hr (*n* = 3) were truncated at 240 hr. Sample processing was initiated within 30 min of collection. Ejaculate volumes were estimated by specimen weight, assuming a semen density of 1.0 g/mL. Sperm concentration was evaluated by hemocytometer (Improved Neubauer; Hauser Scientific Inc., Horsham, PA, USA). Two chambers of the hemocytometer were counted, and the average was used in this analysis. Motility was analyzed using World Health Organization (WHO 1999) criteria; the percentage of all sperm that were classified as forward motile (“A + B,” where highly or moderately progressive sperm are scored as “A” and slow or sluggish progressive sperm are scored as “B”) were used in all analyses and considered motile in this analysis. We also calculated the total sperm count (volume × sperm concentration) and the total motile count (volume × sperm concentration × percent motile). Smears for morphology were made, air-dried, fixed, and shipped to the University Department of Growth and Reproduction at the Rigshospitalet (Copenhagen, Denmark). The slides were Papanicolaou stained and assessed using strict criteria (Menkveld et al. 1990). To increase consistency and comparability of methods over the course of the study, six sets of duplicate semen samples were sent during the study from the University of Copenhagen’s Department of Growth and Reproduction to the Andrology Laboratory (University of Rochester), which is Clinical Laboratory Improvement Amendments certified.

*Physical examination.* A physical examination of each participant was performed, and weight and height assessed, on the same day as semen, urine, and blood sampling. The presence of varicocele or other abnormalities were noted, and testicular size was estimated using Prader’s orchidometer (Andrology Australia, Clayton, Victoria, Australia).

**Figure 1:**
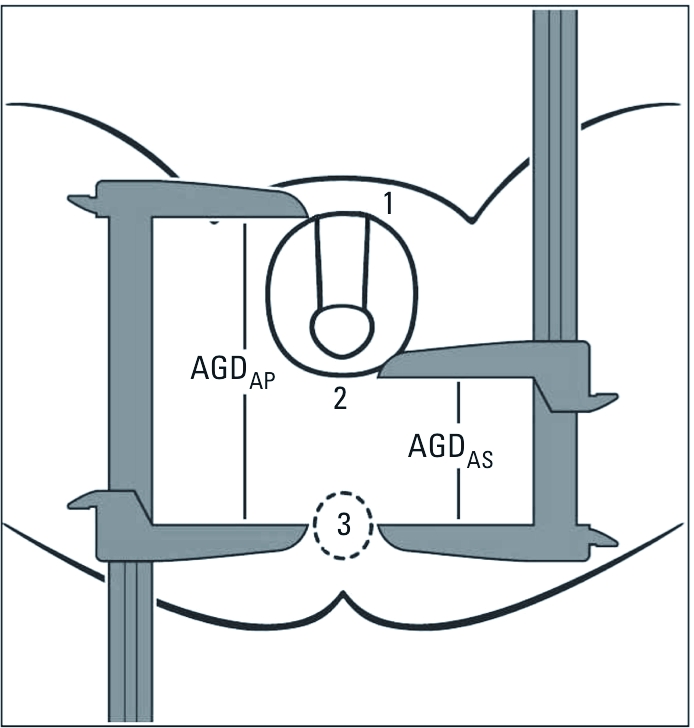
Landmarks for two measurements of AGD: AGD_AP_, from the cephalad insertion of the penis to the center of the anus (point 1 to point 3); and AGD_AS_, from the posterior base (first fold) of the scrotum to the center of the anus (point 2 to point 3). Adapted with permission from Sathyanarayana et al. (2010).

In this study we measured two variants of AGD: The first was measured from the cephalad insertion of the penis to the center of the anus (AGD_AP_; Figure 1, point 1 to point 3), and the second was measured from the posterior base (first fold) of the scrotum to the center of the anus (AGD_AS_; Figure 1, point 2 to point 3). Both were measured using a stainless-steel digital caliper (VWR International, LLC, West Chester, PA, USA) and made while the man was in the lithotomy position, with his thighs at a 45° angle to the examination table. To improve precision, the examiner made each of these measurements twice, and the mean of the two measurements (within-observer mean) was used as the estimate. (More detailed instructions for conducting this exam and an anatomically correct figure demonstrating landmarks are available upon request.)

A single examiner (J.M.) conducted most of the exams (94%), and a second (J. Stevens) examined the remaining seven men. Both examiners independently examined eight of these men in three sessions conducted throughout the collection period. Neither the examiners nor the support staff had knowledge of the men’s semen quality.

*Statistical analyses.* Sperm concentration, total sperm count, and total motile count were logarithmically transformed to normalize their distributions. We examined possible drift in measurements by including exam date in multivariate analyses both as a continuous and as a categorical variable. We assessed within-observer variability in the AGD measurements by calculating the mean absolute difference in measurements. We used multiple regression analyses to identify predictors of each of the two AGD measurements. We then determined the relative importance of these predictors by examining the partial correlations between the measurement and the predictor, controlling for other variables in the model. We also used multiple regression analyses to examine associations between AGD measurements and each semen parameter. Covariates initially examined, both as predictors of AGD measurements and as predictors of semen parameters, were ethnicity, height, body mass index (BMI), examiner, smoking status (current smoker vs. not current smoker), exam date, testicular volume, and presence of testicular abnormalities (varicocele and hydrocele). We also initially included a variable reflecting the number of stressful life events (Dohrenwend et al. 1978), previously shown to be significantly related to sperm count and motility (Gollenberg et al. 2010). When inclusion of a potential covariate resulted in a change in the β-coefficient of < 10%, the variable was not retained in final models. The exception was recruitment period, which was retained even though it had little effect on the regression coefficients for all sperm parameters. In addition, abstinence time was entered into all models predicting sperm concentration, volume, total sperm count, and total motile count, and time from sample collection to sample analysis was included in models predicting sperm motility and total motile count, because these variables are commonly controlled in andrology research. In addition, we examined AGD in relation to the likelihood that a man’s sperm concentration fell below 20 × 10^6^/mL (WHO 1999) using logistic regression and controlling for the same covariates. Final models are described in “Results.” Level of statistical significance was set at 0.05. Once models were determined, two analysts (J.M. and F.L.) conducted these analyses independently using SAS (version 8; SAS Institute Inc., Cary, NC, USA) and SPSS (version 18.0; SPSS Inc., Chicago, IL, USA).

## Results

The RYMS study population was quite homogeneous. Participants were 18–22 years of age (median age, 19.4 years), predominantly Caucasian (81%), nonsmokers (73%), with a median BMI of 24.2. Median sperm concentration was 53.5 × 10^6^/mL, and median total sperm count was 157 × 10^6^. Demographic and reproductive parameters are summarized in Table 1. Although this was a population of apparently healthy young men, 24.6% of them had a sperm count < 20 × 10^6^/mL, a commonly used cutoff for subfertility (WHO 1999).

**Table 1:** Characteristics of RYMS participants (*n* = 126).

Table 1. Characteristics of RYMS participants (*n* = 126).				
Variable		Mean ± SD, or percent		Median (IQR)
Age (years)		19.7 ± 1.0		19.4 (18.8–20.3)
BMI (kg/m^2^)		24.6 ± 3.5		24.2 (22.5–25.8)
Abstinence time (hr)		92.7 ± 78.3		70.7 (61.4–98.6)
Time to start semen analysis (min)		14.0 ± 7.1		10.0 (10.0–15.0)
Testicular volume (mL)		28.7 ± 4.9		26.9 (26.3–33.8)
AGD				
AGD_AS_ (mm)		51.3 ± 14.5		51.7 (43.1–61.1)
AGD_AP_ (mm)		128 ± 13.0		126 (118–135)
Semen parameters				
Seminal volume (mL)		3.3 ± 1.6		3.1 (2.1–4.3)
Sperm concentration (10^6^/mL)		72.6 ± 66.5		53.5 (19.8–99.3)
Percent motile sperm (A + B)*a*		57.4 ± 15.5		60.3 (49.3–69.0)
Percent normal morphology (strict)*b*		8.4 ± 4.6		8.5 (5.0–12.4)
Total sperm count (10^6^)		241 ± 269		157 (66.6–321)
Total motile count (10^6^)*a*		143 ± 155		98.7 (30.5–197)
Ethnicity (%)				
Caucasian		81.0		
African-American		5.6		
Other		13.4		
Current smokers (%)		27.0		
Varicocele present (%)		11.9		
Abbreviations: IQR, interquartile range. **a**One man with long time to analysis was excluded from motility analyses (*n* = 125). **b**Two men with no morphology analysis were excluded (*n* = 124).				

**Figure 2:**
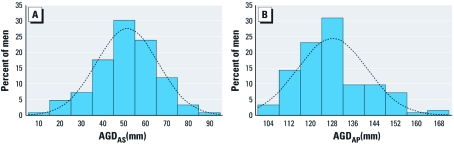
Frequency distributions of AGD_AS_ (*A*) and AGD_AP_ (*B*) in our study population.

The distributions of both AGD_AS_ and AGD_AP_ were approximately normal (Figure 2). AGD_AS_ (mean, 51.3 mm; median, 51.7 mm) was, on average, 40% as long as AGD_AP_ (mean, 128 mm; median, 126 mm), and the SDs of these two measures were similar (14.5 mm and 13.0 mm for AGD_AS_ and AGD_AP_, respectively) (Table 1). As expected, AGD_AS_ and AGD_AP_ were highly correlated [Pearson correlation (*R*) = 0.60, *p* < 0.0001].

*Variability of AGD measurements.* Variation with period of examination. Here we refer to fall 2009 as the first recruitment period and spring 2010 as the second recruitment period. We observed a small but significant decrease in both AGD_AS_ and AGD_AP_ between the first recruitment period (*n* = 44) and the second (*n* = 82). Mean AGD_AS_ was 56.6 mm and 48.5 mm, and mean AGD_AP_ was 132 mm and 126 mm, for men recruited during period 1 and period 2, respectively. Mean age, BMI, abstinence time, and all semen parameters were similar in the two recruitment periods. Despite these differences between time periods, we saw no significant time trend within periods. Although neither exam date nor recruitment period was associated with any of the semen parameters (all *p*-values > 0.24), we retained recruitment period in all final models.

Within- and between-examiner variability. The mean (absolute) difference within examiners was 1.39 mm for AGD_AS_ (2.7% of mean AGD_AS_) and 2.62 mm for AGD_AP_ (2.1% of mean AGD_AP_). We used a mixed model to estimate the interclass correlations, which were 0.91 [95% confidence interval (CI), 0.79–0.97] and 0.95 (95% CI, 0.89–0.98) for AGD_AS_ and AGD_AP_, respectively. We repeated all regression analyses omitting the seven subjects measured by J. Stevens, and results were unchanged.

*Predictors of AGD_AS_ and AGD_AP_.* We examined variables that predicted AGD_AS_ and AGD_AP_ (Table 2). BMI, height, and recruitment period (fall 2009 vs. spring 2010) were significant predictors of both measures. Testicular volume, testicular abnormalities, stress, and ethnicity were not significantly related to AGD, so we did not retain them in the final models.

**Table 2:** Predictors of AGD_AS_ and AGD_AP_ in multivariate models.

Table 2. Predictors of AGD_AS_ and AGD_AP_ in multivariate models.										
		AGD_AS_					AGD_AP_			
Variable		β-Coefficient	*p*-Value	*R*^2^ for single variable	Percent *R*^2a^		β-Coefficient	*p*-Value	*R*^2^ for single variable	Percent *R*^2a^
Height (cm)		0.76	< 0.0001	0.13	51.9%		0.59	< 0.0001	0.09	20.2%
BMI (kg/m^2^)		0.97	0.004	0.06	22.3%		2.18	< 0.0001	0.34	73.3%
Period*b*		–7.70	0.002	0.06	25.8%		–4.75	0.01	0.03	6.5%
Adjusted *R*^2^		0.23					0.45			
**a***R*^2^ for single variable divided by adjusted *R*^2^ for full model. **b**Period of study (fall 2009 vs. spring 2010).										

The model fit was better for predicting AGD_AP_ than AGD_AS_ (adjusted *R*^2^ = 0.45 and 0.23, respectively). BMI accounted for most of the variability in AGD_AP_ but little of the variability for AGD_AS_, whereas height was more influential for AGD_AS_.

*AGD and other covariates in relation to semen parameters.* Covariates retained in final models predicting semen parameters were AGD measures, height, recruitment period, ethnicity (African American or not), abstinence time, and time to sample analysis as described in “Materials and Methods.”

**Figure 3:**
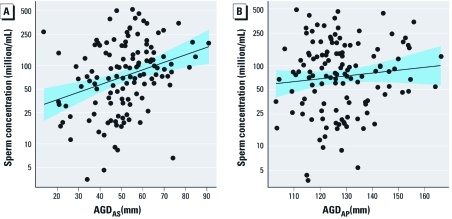
Partial regression plot (mean ± SE) of sperm concentration modeled as a function of (*A*) AGD_AS_ and (*B*) AGD_AP_.

AGD_AS_ was positively related to sperm concentration, motility, morphology, total sperm count, and total motile count (*p*-values 0.002, 0.028, 0.048, 0.006, and 0.009, respectively; Table 3). The associations between AGD_AP_ and sperm count and concentration were negligible, although in a similar direction as those for AGD_AS_. Regression coefficients for the two AGD measures as predictors of sperm motility and morphology were not inconsistent, although CIs for AGD_AP_ were wider and consistent with no association. The residual plots for sperm concentration in relation to AGD_AS_ and AGD_AP_ from our multivariate models, are shown in Figure 3.

**Table 3:** Multivariate analysis for men’s semen parameters and AGD_AS_ and AGD_AP_.*a*

Table 3. Multivariate analysis for men’s semen parameters and AGD_AS_ and AGD_AP_.*a*								
		AGD_AS_				AGD_AP_		
Semen parameter		β-Coefficient	95% CI	*p*-Value		β-Coefficient	95% CI	*p*-Value
Seminal volume (mL)		–0.002	–0.022 to 0.018	0.842		–0.010	–0.032 to 0.011	0.343
ln [sperm concentration (million/mL)]		0.022	0.008 to 0.036	0.002*		0.008	–0.007 to 0.023	0.290
Percent motile sperm (A + B)*b*^,c^		0.227	0.025 to 0.429	0.028*		0.161	–0.055 to 0.379	0.142
Percent morphologically normal sperm*d*		0.061	0.0005 to 0.122	0.048*		0.051	–0.015 to 0.117	0.128
ln [total sperm count (million)]		0.021	0.006 to 0.037	0.006*		0.004	–0.012 to 0.021	0.596
ln [total motile sperm (million)]*b*^,c^		0.024	0.006 to 0.041	0.009*		0.009	–0.010 to 0.028	0.366
β-Coefficient indicates change in semen parameter associated with a 1 mm change in AGD. **a**Controlling for height, ethnicity (African American vs. not), period of study (fall 2009 vs. spring 2010), and ejaculation abstinence time. **b**Also controlling for time from semen collection to start of semen analysis. **c ***n *= 125; one man with long time to analysis was excluded from motility analyses. **d***n *= 124; two men without morphology were excluded. *p < 0.05.								

We also examined sperm concentration dichotomized at 20 × 10^6^/mL (subfertile vs. normal) in relation to AGD, controlling for the same covariates used in the linear regression models. AGD_AS_ was significantly related to this outcome. The risk of subfertility was increased 7.3 times (95% CI, 2.5–21.6) for an (adjusted) AGD_AS_ below the median, compared with AGD_AS_ above the median. Having a low sperm concentration (< 20 × 10^6^/mL) was inversely related to AGD_AS_ (*p* < 0.0019). AGD_AP_ was not related to this outcome.

We calculated the expected change in semen parameters associated with an interquartile increase in AGD_AS_ for a typical study participant. When AGD_AS_ is 43.1 mm, the 25th percentile of the AGD distribution, the expected sperm concentration, using our final regression model, is 34.7 × 10^6^/mL. When AGD_AS_ is 61.1 mm (the 75th percentile), the expected sperm concentration is 51.6 × 10^6^/mL, whereas the predicted value for the 50th percentile of AGD_AS_ is 42.0 × 10^6^/mL. Thus, an interquartile increase in AGD_AS_ is associated with an increase in sperm concentration that is 40.2% of the median, based on the best-fitting model. Similar increases are seen for other sperm parameters, although a smaller increase is seen for percent morphologically normal sperm.

Height, BMI, and time period were all associated with AGD and included in the final models, although none of these variables was associated with any semen parameter in this population. All sperm parameters were significantly lower in the small subgroup (*n* = 7) of African-American men compared with other men in this population (*p*-values for sperm parameters, < 0.001 to 0.016).

## Discussion

This is the first study to measure AGD in adult men and examine the relationships between AGD measures and sperm parameters. We observed significant positive associations between AGD_AS_ and sperm concentration, motility, morphology, total sperm count, and total motile count. The associations we observed between these sperm parameters and AGD were stronger than those for most covariates known to be associated with semen quality. For example, the increase in sperm concentration associated with an interquartile increase in AGD_AS_ is twice as large as that expected in this population to be associated with an interquartile increase in abstinence time (8.4 × 10^6^/mL), a known strong predictor of sperm concentration. Moreover, a man with an AGD_AS_ below the median was 7.3 times as likely to have a sperm concentration in the subfertile range (< 20 × 10^6^/mL) as a man with an AGD_AS_ above the median. This underscores the clinical implications of the associations that we are reporting.

AGD measurements were well tolerated by all subjects and quick to perform, with acceptable intraexaminer reliability. Unlike steroid hormones and semen parameters, AGD measurements are not likely to be sensitive to physiological and lifestyle factors (stress, abstinence time, fever, smoking, etc.) and so may need to be controlled only for body size, as was the case in our study. Therefore, if our results are confirmed, AGD may provide a useful adjunct to these traditional measures of male reproductive function.

*Alternative measures of AGD.* AGD has long been measured in animal studies, but the difference between AGD_AS_ and AGD_AP_ is not readily apparent in newborn pups, although it is clear in humans (Figure 1). AGD has only recently been measured in epidemiological studies, and methods for its reliable measurement are still being developed. Several alternative measurements have been used in examining AGD in human male infants. Thankamony et al. (2009) and Salazar-Martinez et al. (2004) used AGD_AS_, whereas Sathyanarayana et al. (2010) measured AGD_AP_. Romano-Riquer et al. (2007) used a third measure (posterior base of the penis to the anus), in addition to AGD_AS_ and AGD_AP_. Torres-Sanchez et al. (2008) introduced a new measure (the distance from the tip of the coccyx to the center of the anus).

In our previous analyses we measured both AGD_AS_ and AGD_AP_ in human infants and related these to phthalate metabolites in maternal prenatal urine (Swan 2008; Swan et al. 2005). We found inverse associations that were, for most phthalate metabolites, stronger with AGD_AP_ than with AGD_AS_. The distance covered by AGD_AP_ is influenced by penile width and scrotal size as well as AGD_AS_ (Figure 1). Penile width and testicular descent were themselves inversely associated with some phthalate concentrations in infants (Swan 2008), as they are in rodents (Barlow et al. 2004; Gray et al. 2006). Therefore, it is possible that the associations between infant AGD and phthalate metabolite concentrations are stronger for AGD_AP_ than for AGD_AS_.

Because AGD varies with body size, this must be controlled in analysis. Methods for doing this have varied. The anogenital index (AGI; AGD divided by weight) was proposed by Vandenbergh and Huggett (1995) as a way to adjust AGD for body size in newborn mice. Since then, various functions of weight have been proposed in the calculation of this index, including cube root of weight (Gallavan et al. 1999) and weight at weaning (Hotchkiss et al. 2004). Swan et al. (2005) used AGI (AGD/weight at exam) at the mean age of 12.8 months, but this method did not completely remove the effect of weight. Therefore, subsequent analysis (Swan 2008) used weight percentile for age (see Centers for Disease Control and Prevention 2010), a quantity that is largely independent of weight and age and that eliminates confounding by weight. Huang et al. (2009) used AGD, AGD ÷ birth weight, and AGD ÷ birth length. In RYMS men, we found that BMI and height were both significantly associated with AGD_AS_ and AGD_AP_, and we included both measures in our models predicting AGD.

In the present study, we examined men’s sperm parameters in relation to two variants of AGD: AGD_AS_ and AGD_AP_. We saw significant associations with sperm parameters only for AGD_AS_ (Table 3, Figure 3). This may in part be due to the strong influence of BMI on adult AGD_AP_ (Table 2), a quantity that influences the size of the fat pad anterior to the pubic symphysis, an area that is included in AGD_AP_ but not in AGD_AS_. It may also be, however, that different AGD measurements better reflect androgen exposures at different life stages. Once a substantial body of normative data has been accumulated in infants and adults, it should be possible to identify the most androgen-sensitive measure (or measures) and determine which are most strongly related to adult sexual function in adults. Until then, we suggest that future studies continue to collect data on multiple measures.

*Limitations.* Our population was small and limited in age and ethnicity and thus cannot provide normative values for AGD measurements. We saw some differences in AGD and semen quality by race, but numbers were too small to study this adequately. Further, we obtained independent measurements by two examiners on only eight men, too few to adequately estimate inter-rater reliability. Additionally, we noted a small but systematic change in AGD measurements between fall 2009 and spring 2010. Although semen parameters in this population did not vary by study period and period did not confound our primary associations, these data suggest possible measurement drift and the need for ongoing quality control, including frequent replicate measurements by independent examiners throughout the course of any future study.

This is the first study in the United States to report on semen quality in young, unselected men. We therefore cannot assess the representativeness of our study population. There are, however, several studies in Europe that evaluated semen quality in men at the time of screening for military service (Jørgensen et al. 2002). Median sperm concentration in our population was 53.5 × 10^6^/mL, comparable to that seen in young men in these European countries (44–62 × 10^6^/mL).

We measured testicular volume with a Prader orchidometer. We saw significant associations between testicular volume and all semen parameters except motility, but not between testicular volume and either measure of AGD (data not shown). We could not determine whether this is a result of the relatively coarse measurements available with the orchidometer or whether AGD is not correlated with testicular volume. The distribution of our testicular volume measurements appeared to suggest a tendency to report volume in whole numbers (digit preference) and was clearly not normally distributed. Possibly an ultrasound measurement of testicular volume would answer this question.

Our study participants provided only a single semen sample. However, an earlier study of semen quality in 697 men, most of whom provided two samples, determined that after adjusting for important covariates, it made little difference in epidemiological studies whether the analysis includes men who give one semen sample or two (Stokes-Riner et al. 2007).

Finally, we plan to assess reproductive hormones in a future study. A finding of higher follicle-stimulating hormone and/or low inhibin-B or free testosterone in men with shorter AGD would lend support to the association between AGD and semen variables we report here.

## Conclusions

Here we report data showing that one measure of AGD is strongly associated with multiple semen parameters, suggesting AGD’s potential use as a biomarker of developmental antiandrogen exposure. As animal studies (Welsh et al. 2008) have clearly shown, AGD is determined within a discrete masculinization programming window that is determined by androgen action. Thus, the confirmation we present here is highly plausible, because, to date, all key relationships shown for AGD in the rat have also been shown in humans.

If AGD (adjusted for body size) is determined prenatally in humans as in rodents, a shorter AGD in adulthood should reflect a shorter AGD at birth, which in turn reflects decreased androgen exposure *in utero*. Thus, both poorer semen quality and shorter AGD in adulthood may reflect a common origin, including a disruption of testicular development *in utero*, as suggested by the testicular dysgenesis syndrome (TDS) hypothesis (Skakkebaek et al. 2001). As hypothesized, this syndrome, although potentially multifactorial, may be caused by exposure to endocrine-disrupting chemicals during the masculinization programming window (Scott et al. 2008). The increasing incidence of male reproductive disorders (Sharpe and Skakkebaek 2008; Skakkebaek et al. 2001; Toppari et al. 1996) and decreasing sperm counts and testosterone levels (Andersson et al. 2007; Carlsen et al. 1992; Swan et al. 1997; Travison et al. 2007) in many Western countries lend support to this hypothesis. Whether shorter AGD in RYMS men reflects such dysgenesis and whether this is a consequence of fetal antiandrogen exposure are speculative. However, the data we present here, together with our prior study relating shorter AGD to antiandrogenic phthalate exposure in infants, support that interpretation.

We suggest that a shortened male AGD may be an important marker of human TDS. An extended follow-up of a large cohort in which AGD is measured in infancy would be definitive, but logistically challenging. However, confirmation in larger and more diverse populations and in studies of AGD in men with clinical manifestations of TDS (infertile men, those born with cryptorchidism or hypospadias, or men with testicular cancer) would provide persuasive evidence that androgen action during early fetal life exerts a fundamental influence on adult sperm counts in humans, as has been demonstrated in rodents.
